# The complete chloroplast genome of *Elsholtzia byeonsanensis* M. Kim, an endemic species in Korea

**DOI:** 10.1080/23802359.2022.2149248

**Published:** 2022-12-12

**Authors:** Yun-Chang Jeon, Jin-Hyub Paik, Narae Yun, Sangho Choi, Yoonkyung Lee

**Affiliations:** a Laboratory of Plant Systematics, Dept. of Biology, Kyung Hee Univ, Seoul, Republic of Korea; b International Biological Material Research Center, Korea Research Institute of Bioscience and Biotechnology, Daejeon, Republic of Korea

**Keywords:** *Elsholtzia byeonsanensis*, Endemic species, Lamiaceae, Chloroplast genome, Phylogenetic analysis

## Abstract

*Elsholtzia byeonsanensis* M. Kim. is an endemic species in Korea, and its leaves are distinguished from other taxa of *Elsholtzia* by the leathery texture. In this study, we first presented the complete chloroplast genome of *E. byeonsanensis*. The complete chloroplast genome was 150,628 bp, including a large-single copy region (LSC) of 82,738 bp, a small-single copy region (SSC) of 17,492 bp, and a pair of inverted repeat regions (IRs) of 25,199 bp. It contained 112 genes including 78 protein-coding genes, four rRNA, and 30 tRNA genes. The phylogenetic analysis indicated that *E. byeonsanensis* and *E. splendens* formed a monophyletic clade and showed a close relationship. The complete chloroplast genome of *E. byeonsanensis* will provide useful information for phylogenetic and evolutionary studies.

The genus *Elsholtzia* Willd. (Lamiaceae) is primarily distributed in the temperate regions of the Northern hemisphere (Harley et al. 2004), the center of the greatest diversity in the genus is in eastern Asia, particularly China, Korea, and Japan, with approximately 40 species (Li and Hedge 1994, Govaerts et al. 2022). *Elsholtzia byeonsanensis* M. Kim 2012, an endemic species in Korea, is distinguished from other species in *Elsholtzia* by several characteristics; coriaceous leaf textures, petioles glabrous, the adaxial surface of leaf blade glabrous, bract surface glabrous, and coastal habitat (Choi et al. 2012). Here, we report the complete chloroplast genome of *E. byeonsanensis* and investigated its phylogenetic position with the related taxa in the family Lamiaceae.

The genomic DNA used for the analysis was extracted from fresh leaves of *E. byeonsanensis* (35° 39′ 22.09″N, 126° 29′ 41.14″E; Byeonsan-myeon, Buan-gun, Jeollabuk-do, Korea) using modified CTAB method (Healey et al. 2014). The voucher specimen (voucher number KRIB 0090053, Jin-Hyub Paik, jpaik@kribb.re.kr) was deposited in the International Biological Material Research Center (IBMRC), Korea Research Institute of Bioscience & Biotechnology (KRIBB). *E. byeonsanensis* is not an endangered or protected species, and not required specific permission to collect this species. Additionally, ethics approval for this study was obtained by KRIBB Initiative Program (no. KGM4582221). The raw reads were obtained by Illumina HiseqXten platform (San Diego, CA), and were filtered on Trimmomatic v.0.36 (Bolger et al. 2014). The filtered reads were assembled and mapped to a reference chloroplast genome of *E. densa* (accession number: MN793319) using Geneious Prime (v.2021.1.1; Kearse et al. 2012). The chloroplast genome was annotated using Geneious Prime (v.2021.1.1; Kearse et al. 2012) and GeSeq (Tillich et al. 2017) with a chloroplast genome of *E. densa* as a reference. Finally, the complete chloroplast genome of *E. byeonsanensis* has beensubmitted GenBank with accession number: ON040655.

The total length of the complete chloroplast genome of *E. byeonsanensis* in this study has 150,628 bp, which contains a large-single copy region (LSC) of 82,738 bp, a small-single copy region (SSC) of 17,492 bp, and a pair of inverted repeat regions (IRs) of 25,199 bp. The chloroplast genome has 112 genes, including 78 protein-coding genes, four rRNA, and 30 tRNA genes.

The phylogenetic analysis included 15 representative taxa of Lamiaceae based on a previous study (Li et al. 2017), and these sequences were obtained from GenBank. *Caryopteris forrestii* and *Lamium galeobdolon* were selected as outgroup taxa, and all sequences including *E. byeonsanensis* determined in this study were aligned using MAFFT alignment v.7 (Katoh & Standley 2013; http://mafft.cbrc.jp/alignment/server/index.html). The best model TVM + F + I + G4 was calculated by ModelFinder module (Kalyaanamoorthy et al. 2017) in IQ-tree v.1.6.3 (Nguyen et al. 2015), and IQ-tree v.1.6.3 (Nguyen et al. 2015) was used to construct the maximum likelihood (ML) tree.

A ML tree (Figure 1) indicated that five taxa (*E. rugulosa*, *E. byeonsanensis*, *E. splendens*, *E. densa* and *E. densa* var. *ianthina*) of *Elsholtzia* formed a monophyletic clade with 87% bootstrap support value (BS). *E. byeonsanensis* and *E. splendens* formed a monophyletic clade with a highly supported value (BS = 100%), and these species showed a close relationship. *Elsholtzia* was sister to a monophyletic clade of *Perilla* at the intergeneric level (BS = 100%), and the tribe Elsholtzieae including both genera formed a monophyletic clade (BS = 100%). In this study, we first report the complete chloroplast genome of *E. byeonsanensis*, as an endemic taxon in Korea. The results contribute to the conservation of this species and the phylogenetic study of *Elsholtzia.*

**Figure 1.**
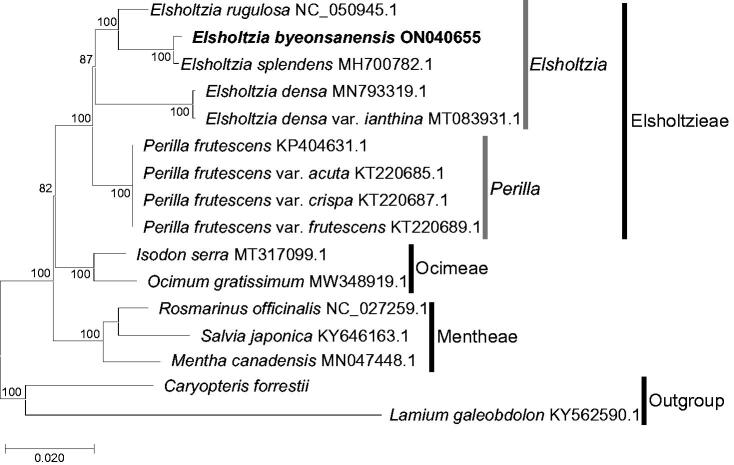
A maximum likelihood tree based on a chloroplast genome of *Elsholtzia byeonsanensis* and related taxa in Lamiaceae. The numbers near the nodes indicate bootstrap support values.

## Data Availability

The genome sequence data that support the findings of this study are openly available in GenBank of NCBI at [https://www.ncbi.nlm.nih.gov] under the accession no. ON040655. The associated BioProject, SRA, and Bio-Sample numbers are PRJNA791132, SRR18450390, and SAMN26865087 respectively.

## References

[CIT0001] Bolger AM, Lohse M, Usadel B. 2014. Trimmomatic: a flexible trimmer for Illumina sequence data. Bioinformatics. 30(15):2114–2120. 24695404 10.1093/bioinformatics/btu170 PMC4103590

[CIT0002] Choi C, Han K, Lee J, So S, Hwang Y, Kim M. 2012. A new species of *Elsholtzia* (Lamiaceae)*: e. byeonsanensis* M. Kim. Korean J Pl Taxon. 42(3):197–201. in Korean).

[CIT0003] Govaerts R, Paton A, Harvey Y, Navarro T. 2022. World checklist of Lamiaceae. Facilitated by the Royal Botanic Gardens, Kew. Published on the Internet; http://apps.kew.org/wcsp/qsearch.do;jsessionid=8491CBBFBD4CE943DA65E5C6F64C0821. (accessed January 2022).

[CIT0004] Harley RM, Atkins S, Budantsev AL, Cantino PD, Conn BJ, Grayer R, Harley MM, De Kok R, Krestovskaja T, Morales R, et al. 2004. Labiatae., in: kadereit JW (Eds), The families and genera of vascular plants VII. Berlin: springer; p. 167–275.

[CIT0005] Healey A, Furtado A, Cooper T, Henry RJ. 2014. Protocol: a simple method for extracting next-generation sequencing quality genomic DNA from recalcitrant plant species. Plant Methods. 10(1):21–28. 25053969 10.1186/1746-4811-10-21 PMC4105509

[CIT0006] Kalyaanamoorthy S, Minh BQ, Wong TKF, von Haeseler A, Jermiin LS. 2017. ModelFinder: fast model selection for accurate phylogenetic estimates. Nat Methods. 14(6):587–589. 28481363 10.1038/nmeth.4285 PMC5453245

[CIT0007] Katoh K, Standley DM. 2013. MAFFT multiple sequence alignment software version 7: improvements in performance and usability. Mol Biol Evol. 30(4):772–780. 23329690 10.1093/molbev/mst010 PMC3603318

[CIT0008] Kearse M, Moir R, Wilson A, Stones-Havas S, Cheung M, Sturrock S, Buxton S, Cooper A, Markowitz S, Duran C, et al. 2012. Geneious Basic: an integrated and extendable desktop software platform for the organization and analysis of sequence data. Bioinformatics. 28(12):1647–1649. 22543367 10.1093/bioinformatics/bts199 PMC3371832

[CIT0009] Li HW, Hedge IC. 1994. Lamiaceae. In: wu, Z.Y. and Raven, P.H. (Eds.) Flora of China 17. St. Louis (MO): Science Press, Beijing & Missouri Botanical Garden Press, p. 294–295.

[CIT0010] Li P, Qi ZC, Liu LX, Ohi-Toma T, Lee J, Hsieh TH, Fu CX, Cameron KM, Qiu YX. 2017. Molecular phylogenetics and biogeography of the mint tribe Elsholtzieae (Nepetoideae, Lamiaceae), with an emphasis on its diversification in East Asia. Sci Rep. 7(1):1–12. 28515478 10.1038/s41598-017-02157-6 PMC5435694

[CIT0011] Nguyen LT, Schmidt HA, von Haeseler A, Minh BQ. 2015. IQ-TREE: a fast and effective stochastic algorithm for estimating maximum likelihood phylogenies. Mol Biol Evol. 32(1):268–274. 25371430 10.1093/molbev/msu300 PMC4271533

[CIT0012] Tillich M, Lehwark P, Pellizzer T, Ulbricht-Jones ES, Fischer A, Bock R, Greiner S. 2017. GeSeq–versatile and accurate annotation of organelle genomes. Nucleic Acids Res. 45(W1):W6–W11. 28486635 10.1093/nar/gkx391 PMC5570176

